# Environmental remediation approaches by nanoscale zero valent iron (nZVI) based on its reductivity: a review

**DOI:** 10.1039/d4ra02789b

**Published:** 2024-07-04

**Authors:** Mingyue Liu, Gang Chen, Linli Xu, Zhicai He, Yuyuan Ye

**Affiliations:** a School of Pharmaceutical and Chemical Engineering, Taizhou University Taizhou 318000 Zhejiang Province China liumingyue0820@126.com

## Abstract

The fast rise of organic and metallic pollution has brought significant risks to human health and the ecological environment. Consequently, the remediation of wastewater is in extremely urgent demand and has received increasing attention. Nanoscale zero valent iron (nZVI) possesses a high specific surface area and distinctive reactive interfaces, which offer plentiful active sites for the reduction, oxidation, and adsorption of contaminants. Given these abundant functionalities of nZVI, it has undergone significant and extensive studies on environmental remediation, linking to various mechanisms, such as reduction, oxidation, surface complexation, and coprecipitation, which have shown great promise for application in wastewater treatment. Among these functionalities of nZVI, reductivity is particularly important and widely adopted in dehalogenation, and reduction of nitrate, nitro compounds, and metal ions. The following review comprises a short survey of the most recent reports on the applications of nZVI based on its reductivity. It contains five sections, an introduction to the theme, chemical reduction applications, electrolysis-assisted reduction applications, bacterium-assisted reduction applications, and conclusions about the reported research with perspectives for future developments. Review and elaboration of the recent reductivity-dependent applications of nZVI may not only facilitate the development of more effective and sustainable nZVI materials and the protocols for comprehensive utilization of nZVI, but may also promote the exploration of innovative remediation approaches based on its reductivity.

## Abstract 2

This review summarized recent reports in the reductive environmental remediation by nZVI and gave prospects for future research, which may benefit the exploration of effective and sustainable nZVI materials and innovative remediation approaches.

## Graphical abstract

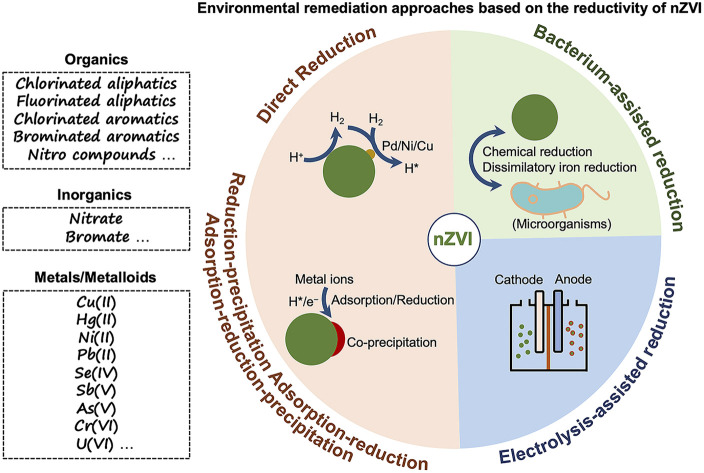

## 1. Introduction

With the growth of population, the development of modern industry, and the accelerating economy, the demand for clean water resources is gradually increasing,^1,2^ while these valuable water resources have been seriously threatened by the growing intractable and unmanageable water pollution, originating from the discharge of industrial, domestic, and agricultural effluents.^3,4^ Wastewater usually contains various containments, including dyes, halogenated compounds, pesticides, antibiotics, metal/metalloid ions, radionuclides, nitrates, and so on, which can bring significant risk to human health and the ecological environment *via* bioaccumulation, antibiotic resistance, carcinogenesis, and ecotoxicological effects.^5,6^ Therefore, the remediation of wastewater is extremely urgent and has received increasing attention.^7–9^

In recent decades, nanoscale zero valent iron (nZVI) and its composites have been employed for wastewater remediation, and performed significant removal capacity of various pollutants, indicating a high potential in practical application.^10,11^ nZVI was first synthesized *via* the reduction of Fe(ii)/Fe(iii) with borohydride in 1995, and adopted in the dechlorination of chlorinated organics in 1997.^12^ From then onwards, the preparation and application of nZVI have been extensively studied.^13,14^ As the fourth most prevalent element and the second most abundant metallic element on the earth, iron is abundant, environmentally friendly and useful for application in environmental remediation.^15,16^ Compared with zero valent iron (Fe^0^), which is a reactive metal with standard redox potential (*E*^0^ = −0.44 V), nZVI has a smaller size (1–100 nm), higher specific surface area, and thus greater reactivity.^17^ The core–shell structure and Fe-containing oxide layer endow nZVI with distinctive reactive interfaces, which provide plentiful active sites for the reduction, oxidation, and adsorption of contaminants.^18,19^ Given these abundant functionalities of nZVI, it has undergone significant extensive studies on environmental remediations, linking to various mechanisms, such as reduction, oxidation, surface complexation, precipitation, which have shown great promise for the treatment of wastewater.

On account of the excellent reducing capability of nZVI, it can reductively transform contaminants in groundwater and industrial wastewaters under anoxic conditions, such as halogenated organics, nitro compounds, nitrate, metal/metalloid ions (Cr(vi), As(v), Ni(ii), and Cd(ii)), and radioactive ions (U(vi) and Tc(vii)) into low-toxic compounds or biodegradable/recyclable forms.^2,19–21^ During the reduction or aqueous storage process, the oxidation of Fe^0^ surface layer by H_2_O and O_2_ can lead to the formation of a unique reactive oxide film on the surface of nZVI, which provides abundant active sites for surface complexation and adsorption of contaminants and their subsequent transformation *via* oxidation or reduction pathways.^16,22^ This inevitable oxidation of Fe^0^ surface generates Fe(ii)/Fe(iii)-containing minerals, endowing nZVI with the functionality of sorption and complexation.^23–25^ Concomitantly, more remediation approaches, such as adsorption–reduction, and adsorption–reduction–precipitation in variably oxic or anoxic environments, have emerged based on the reductivity of nZVI.^19,26^ Moreover, nZVI can be easily recycled with an external magnet, and the recycling is still viable even if the nZVI has undergone surface oxidation or surface passivation.^18,27^ This character endows nZVI with environmental benignancy and convenient recyclability in the applications of remediation or enrichment, necessitating it as a promising candidate for environmental applications.

**Scheme 1:**
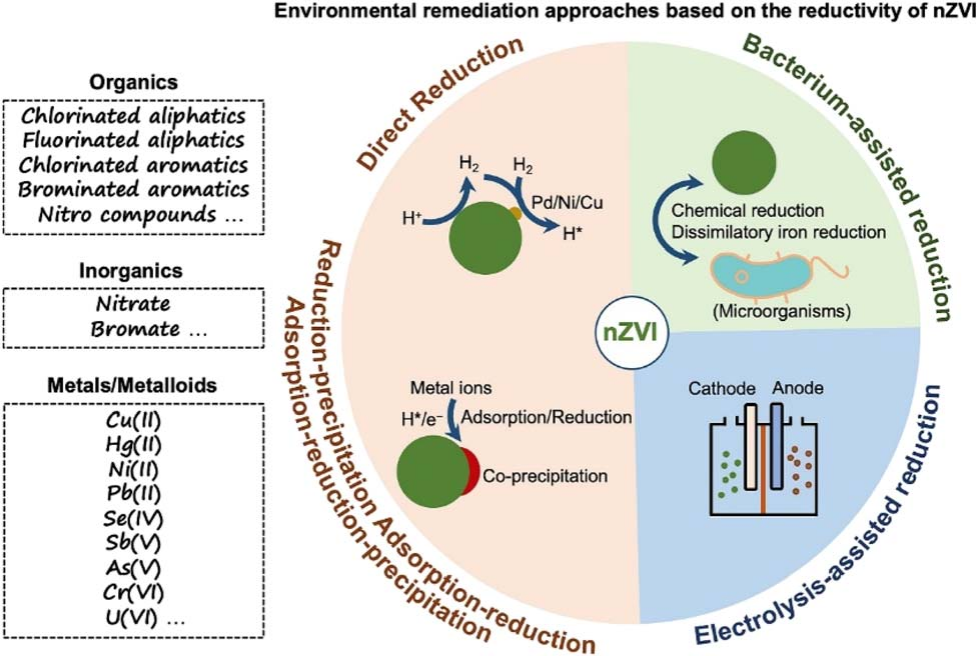
Environmental remediation approaches for various pollutants by nZVI based on its reductivity.

Accordingly, nZVI has attracted great attention and has been widely applied in wastewater remediation based on its reductivity *via* various approaches, such as chemical reduction applications, electrolysis-assisted reduction applications, and bacterium-assisted reduction applications. However, there is a lack of papers that critically analyse the recent advances in the applications of nZVI in environmental remediation based on its reductivity, especially in the comprehensive examination of the multiple progressive environmental remediation approaches based on the reductivity of nZVI. In this regard, this review aims to provide an overview of the recent advances in the reductivity-dependent environmental remediation approaches by nZVI (Scheme 1), and to gain insight into the current progress, deficiencies, and future improvement in the reduction application of nZVI.

## 2. Chemical reduction by nZVI

Remedial efforts employing nZVI *via* reduction have been performed on 1,2-dichloroethylene (DCE),^28^ trichloroethylene (TCE),^29–32^ perchloroethylene (PCE),^28^ 1,1,1-trichloro-2,2-bis(4-chlorophenyl) ethane (DDT),^33^ 2,4-dichlorophenol (2,4-DCP),^34–37^ trichloronitromethane (TCNM),^38^ 1,2,4-trichlorobenzene (1,2,4-TCB),^39^ 2,4,6-trichlorophenol (TCP),^40,41^ 2-chlorobiphenyl (PCB1),^42,43^ diclofenac (DCF),^44^ chlorinated organophosphate esters (Cl-OPEs),^45^ 2,2′,4,4′-tetrabromodiphenyl ether (BDE-47),^46,47^ tetrabromobisphenol A (TBBPA),^48,49^ methylene blue,^50^*p*-nitrophenol,^51,52^ nitrobenzene,^53,54^ mono-brominated diphenyl ether (BDE-3),^55^ Cr(vi),^56–61^ Sb(v),^62^ Ni(ii),^63–66^ Pb(ii),^65,67^ Cu(ii),^68^ As(v),^69^ U(vi),^23,70–72^ NO_3_^−^,^73–76^ and so on.

As nZVI has a large specific surface area and high reactivity, it is prone to be corroded by H_2_O and dissolved O_2_ in solutions, leading to dissolution and surface passivation. The oxidation of nZVI with H_2_O can produce H_2_, which is vital for the reduction of contaminants.^41^ While surface passivation of nZVI can result in deterioration of reductivity, which is a primary obstacle in the reduction application of nZVI.^77^ Moreover, dispersed nZVI particles are susceptible to agglomerate and form chain-like aggregates as a result of magnetism and van der Waals forces, thus giving rise to a decrease in specific surface area and reductive reactivity.^78,79^ Thereby, several strategies have been employed in laboratory research and field trials to overcome these obstacles, such as modifying and emulsifying nZVI, doping nZVI with catalytic noble metal (*i.e.*, Pd, Ni, Cu), and supported nZVI onto solid porous materials.^12,80^ The well-designed functionalized support gives nZVI material with enhanced adsorption capacity and reductivity, triggering a cooperative remediation strategy of adsorption–reduction. In the reductive and adsorption–dechlorination applications, nZVI showed substantially high reactivity and removal efficiency towards organic chlorides.^81,82^ Moreover, supported nZVI materials not only have good adsorption performance towards metal/metalloid ions, but also can reduce them to low valent metal ions or metallic state, hence realizing the removal and enrichment of metal/metalloid ions from aqueous solution *via* magnetic separation.^83,84^

### 2.1. Dechlorination

Chlorinated organics are usually toxic, mutagenic, and carcinogenic to human beings and the ecosystem.^85^ Due to their extensive use and refractory nature, the presence of these compounds in water bodies is almost ubiquitous.^86^ Efficient dechlorination of chlorinated organics by nZVI is highly depend on its reductivity. In a typical dechlorination process, H_2_O is firstly reduced and generates H_2_ (eqn (1)), which is subsequently activated by the doped catalytic metal (*i.e.*, Pd) and forms activated hydrogen atom (H·), executing hydro-dechlorination towards contaminants. Accordingly, nZVI with high reductivity can produce H_2_ at a higher rate, and perform superior dechlorination efficiency.

1 Fe^0^ + 2H_2_O → Fe^2+^ + H_2_↑ + 2OH^−^

#### 2.1.1. Direct dechlorination

**Fig. 1:**
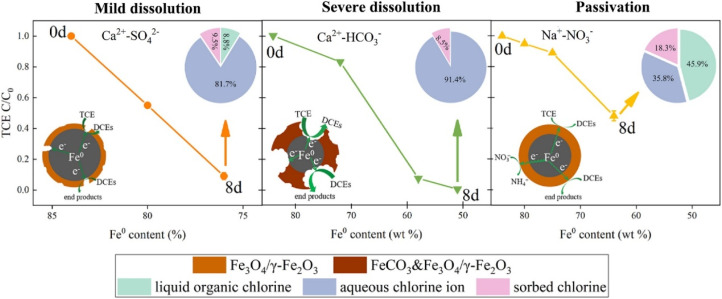
Scheme of three dechlorination processes of TCE caused by diverse anaerobic corrosion mechanisms under the influence of Ca^2+^-HCO_3_^−^, Ca^2+^-SO_4_^2−^ and Na^+^-NO_3_^−^ within 8 days (8d), the insert pie charts (where the arrows point) are the proportional distributions of chlorine species during TCE reduction by nZVI,^29^ copyright 2021 Elsevier.

Direct dechlorination of chlorinated organics *via* hydro-dechlorination has been widely studied, in which Cl atom is replaced with H atom and forms much less toxic hydrocarbons. The application of nZVI in the dechlorination of TCE has been comprehensively studied, and the long-term application of nZVI in the actual water systems has been a research hotspot in recent years.^87^ Yang *et al.* investigated the effect of nZVI anaerobic corrosion on the reductive dechlorination of TCE with various groundwater geochemical constituents (Na^+^, Ca^2+^, SO_4_^2−^, HCO_3_^−^, NO_3_^−^).^29^ This study reveals that different water chemical condition leads to different anaerobic dissolution processes of surface layer and corrosion processes of nZVI (dissolution corrosion), and result in different dechlorination processes of TCE. Specifically, the dissolution corrosion of Ca^2+^-HCO_3_^−^ towards nZVI gives rise to the exposure of Fe^0^ from the passivation layer, which promotes the reductive dechlorination of TCE (Fig. 1). While Ca^2+^-SO_4_^2−^ and Na^+^-NO_3_^−^ have a relatively slight dissolution corrosion effect on nZVI (Fig. 1). In future practical remediation applications of nZVI in contaminated sites, taking the effect of water chemical conditions on nZVI corrosion and dechlorination performance into consideration may be necessary.

**Fig. 2:**
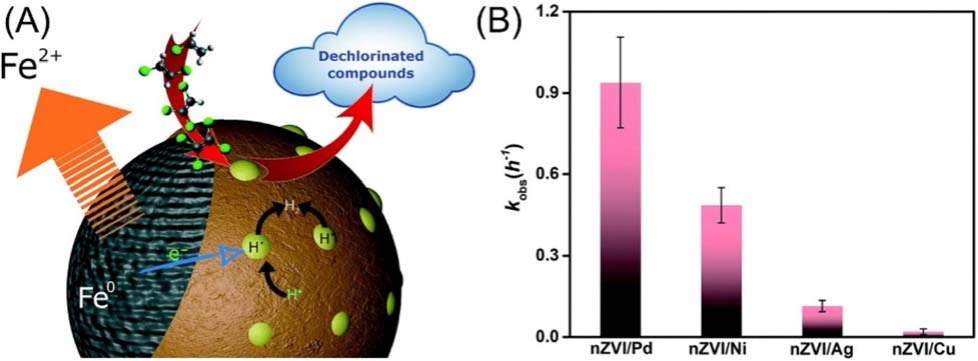
(A) Schematic representation of the surface-mediated catalytic degradation of dechlorinated compounds by nZVI/Pd and nZVI/Ni; (B) comparison of various bimetallic nZVI on dechlorination performance,^28^ copyright 2022 Royal Society of Chemistry.

**Fig. 3:**
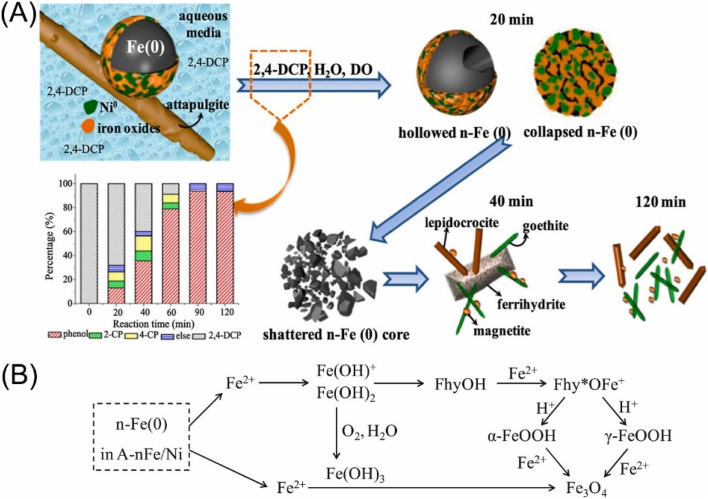
(A) The composition evolution of attapulgite supported nZVI/Ni during the dechlorination of 2,4-dichlorophenol; (B) schematic transformation and compositional evolution of Fe^0^ in nZVI/Ni during dechlorination;^34^ n-Fe(0) refers to nZVI, A-nFe/Ni refers to attapulgite supported Fe/Ni bimetallic nanoparticles, FhyOH refers to ferrihydrite (Fe_5_O_3_(OH)_9_), and Fhy* refers to dehydroxylated ferrihydrite (Fe_5_O_3_(OH)_8_^+^), copyright 2021 Elsevier.

Regarding that the cleavage of aromatic C–Cl bonds is more difficult than that of aliphatic C–Cl bonds driven by the electron transfer process by nZVI, efficient dechlorination of chlorinated aromatic compounds commonly calls for the doping nZVI with Pd or Ni, which provides hydrogenation activity for dechlorination by catalytic transforming H_2_ to H· (Fig. 2A).^28,88,89^ A comparative study conducted by Venkateshaiah *et al.* found that the order of dechlorination efficiency by bimetallic nZVI is: nZVI/Pd > nZVI/Ni > nZVI/Ag > nZVI/Cu (Fig. 2B).^28^ Zhuang *et al.* prepared graphitized carbon supported nZVI/Ni, which performed satisfied dechlorination efficiency on TCP compared with pristine nZVI, owing to the generation of atomic H· and the inhibition of oxidation and deactivation of nZVI by graphitized carbon.^40^ Liu *et al.* employed nZVI/Ni in the dechlorination of TCE, the efficiency of which exhibited 1.4–3.5 times higher than that of nZVI.^31^ A study conducted by Anang *et al.* focused on the transformation and the fate of attapulgite supported nZVI/Ni during the dechlorination, which is vital for the predicting of nZVI surface chemistry, providing guidance for more utilization nZVI, and studying the impact of the corrosion process on environment.^34^ Their research indicates that the Fe^0^ core is firstly hollowed, collapsed, and then gradually formed poorly crystallized Fe_5_O_3_(OH)_9_ at the first dechlorination stage, which later transformed to γ-FeOOH, α-FeOOH, and Fe_3_O_4_ by the end of the dechlorination (Fig. 3). The doping of Ni and the supporting on attapulgite can significantly accelerate the dechlorination and this transformation.

Besides doping, some scholars use enhancement techniques or external artificial intervention measures to improve the effectiveness of nZVI in the dechlorination of chlorinated aromatic compounds. Blundell *et al.* applied ultrasonic energy in the reductive dechlorination of DDT, which prevents the aggregation and surface deactivation of nZVI, leading to a significant increase in the dechlorination efficiency of DDT.^33^ Chi *et al.* capsulated nZVI into Fe_3_O_4_@SiO_2_ to stimulate magnetic spatial confinement effect and change the electron transfer pattern, which realized a controlled interfacial electron transfer behavior and the prevention of formation oxide surface layer on Fe^0^, achieving an increased dechlorination efficiency on TCP of 5.53 times in magnetic field compared with that of pristine nZVI without magnetic field.^41^

**Fig. 4:**
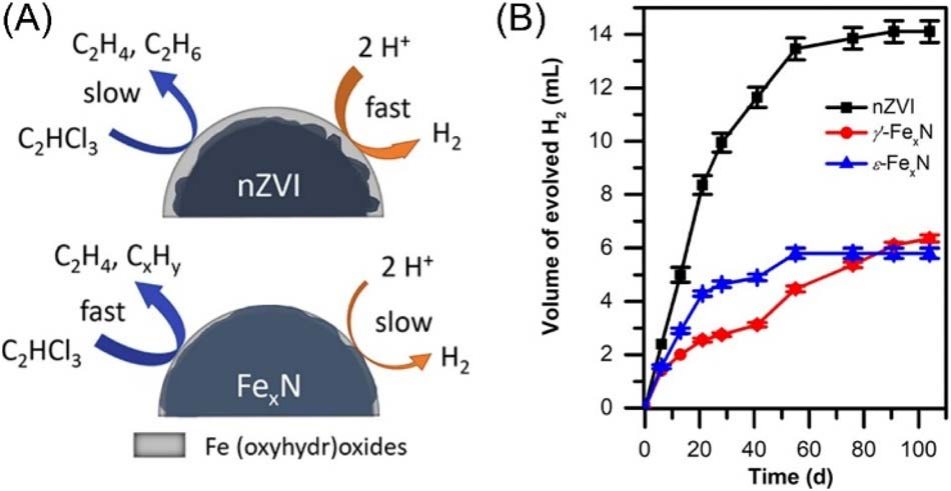
(A) The dechlorination mechanism of pristine nZVI and nitrogen-doped nZVI; (B) hydrogen evolution during aging of pristine nZVI and nitrogen-doped nZVI,^30^ copyright 2022 American Chemical Society.

Despite that H-transfer is a well-recognized dechlorination mechanism, e-transfer between nZVI and target contaminant has also been proposed as a complementary mechanism that may occur in dechlorination without a catalyst. Brumovský *et al.* prepared nitrogen-doped nZVI (Fe_*x*_N) by passing a gaseous NH_3_/N_2_ mixture over nZVI at elevated temperatures to enhance the dechlorination performance of nZVI by changing the hydro-dechlorination mechanism to direct electron transfer mechanism (Fig. 4).^30^ Fe_*x*_N showed a 20-fold increase in the TCE dechlorination rate compared with pristine nZVI and retained high reactivity even after three months of aging. While, its dechlorination performance on more stable chlorinated aromatic compounds is still lack of knowledge.

#### 2.1.2. Adsorption–dechlorination

**Fig. 5:**
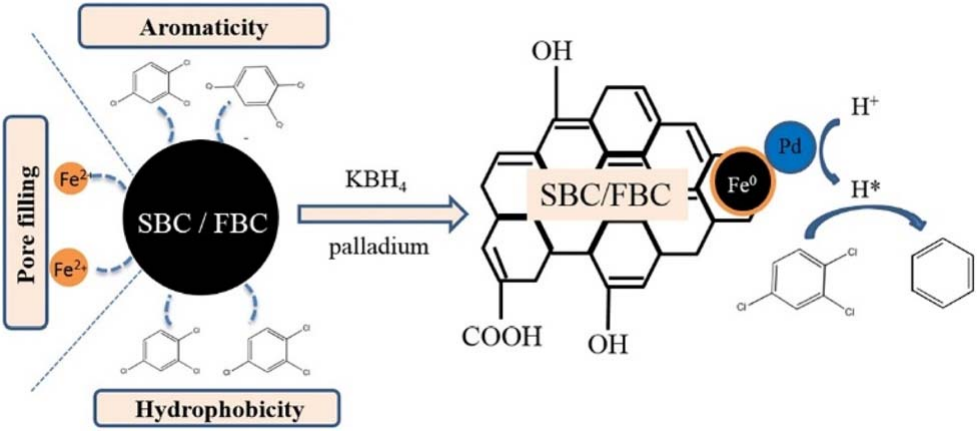
Schematic representation of the adsorption and reductive dechlorination of TCB by modified biochar (with high aromaticity and hydrophobicity) supported nZVI/Pd;^39^ SBC is NaOH-modified biochar and FBC is HF-modified biochar, copyright 2020 Elsevier.

**Fig. 6:**
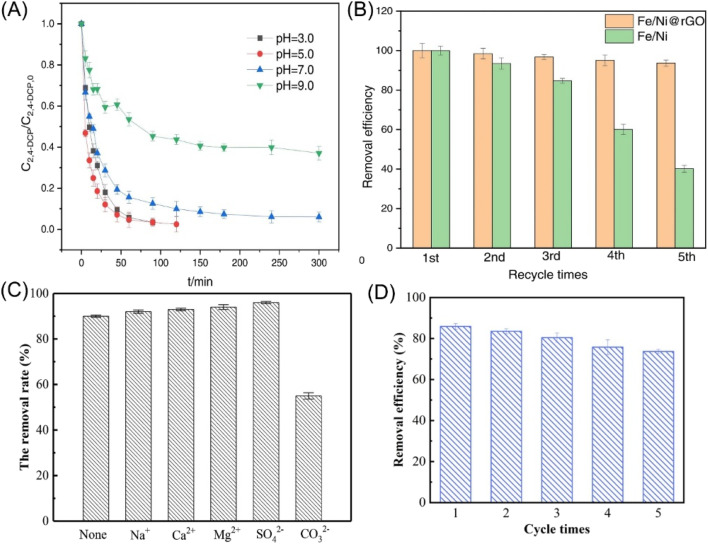
(A) Effect of initial pH of solution on 2,4-DCP removal in nZVI/Ni@rGO system; (B) the reusability of the nZVI/Ni@rGO composites in dechlorination,^36^ copyright 2023 Elsevier. (C) Effect of coexisting ions on the removal efficiency of 2,4-DCP by nZVI/Ni (the concentrations of coexisting ions are all 200 mg L^−1^),^35^ copyright 2021 Elsevier. (D) Reusability of nZVI/Pd@ZIF-8 in 2,4-DCP removal,^37^ copyright 2023 Elsevier.

When supported nZVI onto porous support, coupling the adsorption function of support with the reductive dechlorination of nZVI, provides a novel strategy for dechlorination, which enhances the interfacial dechlorination reaction and improves dechlorination efficiency. Shang *et al.* supported nZVI/Pd in HF or NaOH-modified biochar (FBC-nZVI-Pd, SBC-nZVI-Pd), and demonstrated a significantly enhanced removal efficiency of 98.8% in 48 h toward TCB, attributing to the active sites providing by pore-filling spaces of HF-modified biochar (FBC) or NaOH-modified biochar (SBC) for the adsorption of Fe^2+^ during the preparation of nZVI and the adsorption of TCB during dechlorination (Fig. 5).^39^ The SBC-nZVI-Pd has increased specific surface area, aromaticity, and hydrophobicity after the modification, endowing it with excellent adsorption performance towards organic pollutants (Fig. 5). The study indicated that pollutants are firstly enriched and subsequently dechlorinated. In the first stage (12 h), the proportion of dechlorination is about twice that of adsorption, while the proportion of adsorption is gradually increased as the reaction reaches an equilibrium in the residual stage. Zhang *et al.* modified spent bleaching earth carbon with cetyltrimethylammonium bromide and loaded with nZVI, for the dechlorination of DCF.^44^ This work indicated that the synergistic strong adsorption effect of the material and reduction effect of nZVI lead to a high-efficiency removal of DCF (87%). Huang adopted reduced graphene oxide supported nZVI/Ni for the rapid adsorption (π–π interaction between 2,4-DCP and graphene nanosheet) and dechlorination 2,4-DCP, achieving a removal efficiency of 95% in 3 h.^36^ The composites had good stability and high recycling value, which can be repeatedly used five times without obvious decrease in efficiency (Fig. 6A). The dechlorination always prefers appropriate acidity conditions, which could accelerate the corrosion of nZVI and produce sufficient hydrogen for the subsequent formation of reactive atomic hydrogen by Ni, thus facilitating hydro-dechlorination (Fig. 6B). While, excessive acidic conditions may lead to a fast loss of nZVI/Ni and form excessive hydrogen bubbles at the interface of nZVI/Ni, resulting in diminished dechlorination efficiency on the contrary (Fig. 6B).

**Fig. 7:**
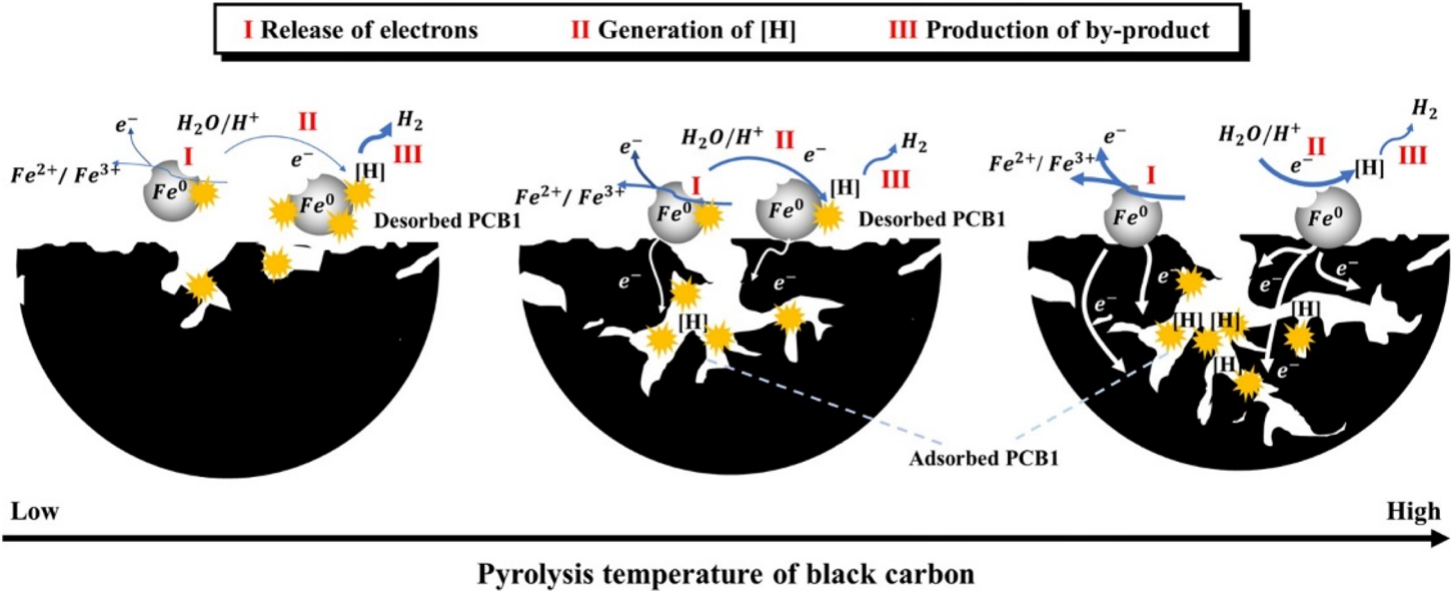
Conceptual diagram for the mechanism of dechlorination of adsorbed PCB1 with different adsorption states regulated by three typical black carbon obtained at different pyrolysis temperatures,^42^ copyright 2021 Elsevier.

Normally, the support is not only adsorbent for pollutants, but also beneficial for the distribution of nZVI and prevention of the aggregation of nZVI owing to the confinement and dispersion effect.^90^ Meanwhile, some scholars reported that biochar can also enhance the conductivity of bimetallic nZVI/Ni, thus promoting the production of dominant reactive atomic hydrogen, and demonstrating remarkable TCE degradation efficiencies of 91.8% in tap water.^21^ A strong adsorption effect may not always beneficial for the dechlorination. Xu *et al.* discovered that the adsorption state of PCB1 on support has a prominent effect on the dechlorination by nZVI/Pd and nZVI/Ni (Fig. 7).^42,43^ As PCB1 is difficult desorption from black carbon from low pyrolysis temperatures, dechlorination is inhibited, the efficiency of which is only 53.5% in 48 h.^42^ While, the electron releasing of Fe^0^ and the generation of H· is enhanced in high temperature black carbon due to its high conductivity, thus reducing the inhibition of adsorption of PCB1 on dechlorination to some extent, achieving an efficiency of 95.3% in 48 h. Their findings suggested that characteristics of support and adsorption state of pollutants may heavily affect the dechlorination efficiency of nZVI.

In addition, polystyrene resin, bentonite, Al-based MOFs (MIL-96), and zinc-based zeolitic imidazolate framework (ZIF-8) have also been employed in the embedding of nZVI. Zhang *et al.* indicated that 2,4-DCP is completely dechlorinated by polystyrene resin supported nZVI/Ni, and the generated phenol can be adsorbed and magnetic separation from water.^35^ As reductive dechlorination prefers acid conditions, the presence of CO_3_^2−^ had a significant inhibition effect on dechlorination (Fig. 6C). Baldermann *et al.* immobilized nZVI on bentonite substrate, forming “micro-reactors” for the sorption of TCE on the clay surface and the subsequent dechlorination.^91^ Bentonite prevents the agglomeration or inactivation of nZVI due to the uptaking of dissolved Fe species during nZVI corrosion in bentonite and the formation of Fe-smectite, suppressing the deterioration of the reactivity of nZVI. MIL-96 supported nZVI provides an efficient adsorption–degradation process for the reduction of TCNM to methylamine.^38^ High concentrations of coexisting ions, such as SO_4_^2−^, PO_4_^3−^, and NO_3_^−^ bring adverse effects on the dechlorination of TCNM; while, the effects of Cl^−^ and CO_3_^2−^ were insignificant. Weng *et al.* employed ZIF-8 supported nZVI/Pd for the removal of 2,4-DCP and achieved a removal efficiency of 85.9% within 120 min owing to the synergism of adsorption and reduction.^37^ Benefitting from the activity protection of nZVI/Pd by ZIF-8, the removal efficiency maintained relatively high at 73.7% even after 5 repeated uses (Fig. 6D).

### 2.2. Other dehalogenation

#### 2.2.1. Debromination

Brominated flame retardants, which have been widely used as additives in circuit boards and plastics to improve flame resistance, are of significant concern to human health owing to their endocrine disruptive risks and immune toxic characteristics.^48,49^ Debromination is vital for the treatment and disposal of organic bromide.

**Fig. 8:**
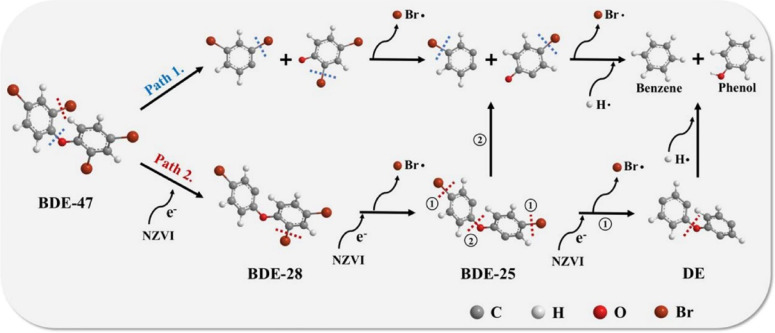
Conceptual debromination pathway of BDE when pyrolysis with nZVI,^47^ copyright 2023 Elsevier.

Chen *et al.* reported the ball milling of nonmetallic particles from waste-printed circuit boards with nZVI, the results of which indicated that the content of bromine on the surface of the nonmetallic particles was reduced by 50%.^92^ The debromination is not only attributed to the electron transferred from nZVI during ball milling, but also the reduced energy required to break the C–Br bond promoted by the stretch and length increase of the C–Br bond after pentabromodiphenyl ether gained electrons from nZVI. Huang *et al.* added nZVI in vacuum low-temperature pyrolysis of resin particles from waste-printed circuit boards and achieved 69% debromination efficiency, which was obviously higher than that without nZVI (20%).^47^ The existence of nZVI changes the degradation pathway of organic bromides by providing electrons and H, transforming organic bromine of resin into fixed inorganic bromine (Fig. 8). In the debromination of BDE-47 without nZVI, the C–O bond may break firstly and the C–Br bonds will break in sequence, and eventually formed phenol and benzene *via* the reaction of two monomers with hydrogen radicals generated from pyrolysis (as shown in path 1.). While, the presence of nZVI in the debromination of BDE-47 will make the C–Br bond more likely to fracture by providing electrons, resulting in a change of bond breaking sequence, that is, the C–Br bonds may break earlier than C–O bond (as shown in path 2.). The following pathway for the debromination of BDE-25 depends on the bond breaking sequence of C–O bond and C–Br bonds in *para*-position. If the C–Br bonds in *para*-position are more likely to break than C–O bond, the debromination reaction will continues, otherwise subsequent debromination will occur in two monomers as shown in path 1. Notably, ∼83% of nZVI can be separated and reused in the debromination.

**Fig. 9:**
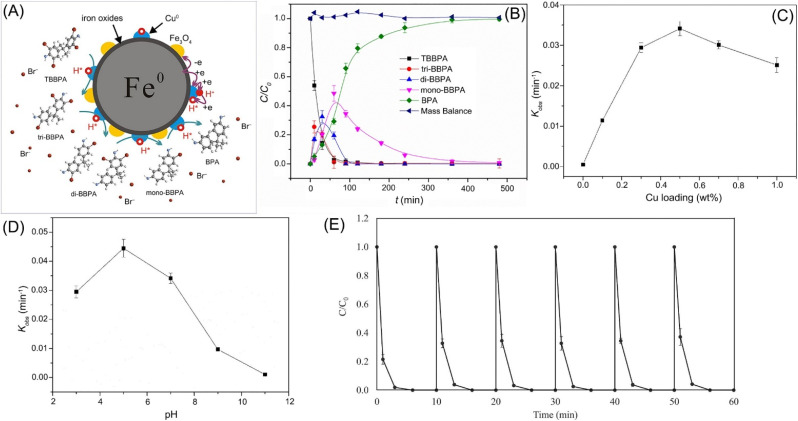
(A) Scheme of the debromination of TBBPA by Cu-nZVI; (B) time-dependent concentration of the intermediates (tri-BBPA, di-BPPA, and mono-BPPA) and final product at pH 7; (C) effect of Cu loading and (D) pH on the debromination kinetics,^42,48^ copyright 2019 Elsevier. (E) The reusable tests of Cu-nZVI in TBBPA debromination,^49^ copyright 2022 Elsevier.

The debromination efficiency by nZVI alone is usually low. To achieve higher efficiency and more complete debromination of organic bromides, Pd, Cu, and Ni are often coated on nZVI surface. Compared with nZVI, the debromination efficiency by Pd-doped nZVI was about 2–4 orders of magnitude greater in the debromination of mono-, di-, and tri-brominated diphenyl ethers.^93^ Li *et al.* employed Cu-nZVI in the batch debromination of TBBPA (10 mg L^−1^), which was mostly transformed to bisphenol A within 240 min (Fig. 9).^48^ While, without doped Cu, there was almost no obvious debromination occurred (Fig. 9C). As low pH is beneficial for the formation of H· and inhabitation of the deactivation of nZVI, the debromination efficiency by Cu-nZVI reached maximum value at pH 5 (Fig. 9D). Huang *et al.* further indicated that catalytic debromination is dominated in the removal of TBBPA in the condition of pH 3–7, high temperature (≥25 °C), and Cu loading; while, adsorption will be dominated in the condition of alkaline condition (pH ≥ 11), low Cu doping, or low temperature (≤15 °C).^49^ Moreover, in the recyclability tests of Cu-nZVI for the debromination of TBBPA, the removal efficiency remained roughly high throughout the experimental cycles, suggesting no obvious substantial drop in catalytic performance (Fig. 9E).

**Fig. 10:**
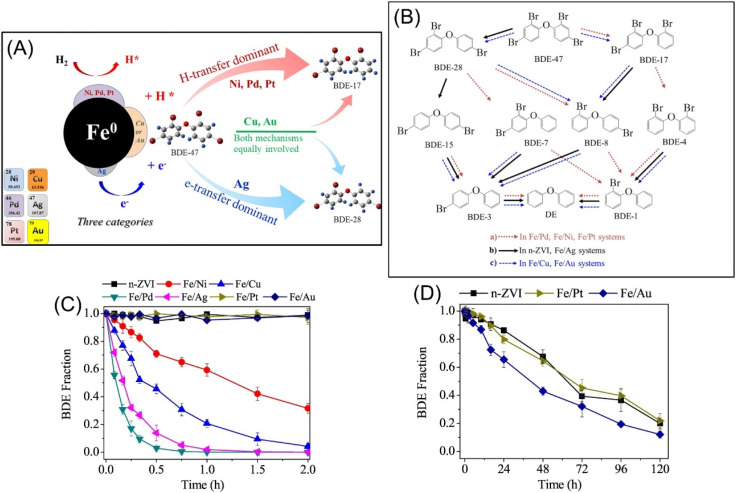
(A) Schematic mechanisms and pathways of debromination of polybrominated diphenyl ethers by Ni/Pd/Pt/Cu/Au/Ag doped nZVI systems; (B) possible debromination pathways of BDE-47 in various bimetallic systems; (C) debromination of BDE-47 in various bimetallic systems in a short period and (D) a long period,^46^ copyright 2019 Elsevier.

As regard to the mechanism of the accelerated dehalogenation by doped nZVI, scholars present H-transfer and e-transfer processes. In the H-transfer mechanism, the doping metal with high standard redox potential acts as a catalyst to improve the dehalogenation, *via* (i) the forming of galvanic cells with nZVI, enhancing Fe^0^ corrosion and H_2_ formation; (ii) catalysing hydrogenation by promoting the transformation of adsorbed H_2_ to H·.^48^ In the e-transfer mechanism, the doping metal forms a galvanic couple with nZVI, enhancing the electron transfer (e-transfer) from nZVI to contaminants, thus enhancing the dehalogenation.^46^ Wang *et al.* conducted experiments to investigate the relative significance of e-transfer and H-transfer in Cu, Ni, Pd, Ag, Pt, and Au doped nZVI in debromination of BDE-47, which primarily revealed that the debromination of BDE-47 by Fe/Ni, Fe/Pd and Fe/Pt follows H-transfer dominant mechanism, the debromination of BDE-47 by Fe/Ag follows e-transfer dominant mechanism, whereas e-transfer and H-transfer mechanisms may be equally involved in the debromination of BDE-47 by Fe/Cu and Fe/Au (Fig. 10A).^46^ The possible debromination pathways of BDE-47 in various bimetallic systems are summarized in Fig. 10B. In the e-transfer process, *ortho*-bromine of BDE-47 is preferentially debrominated and generate BDE-28, whereas in the H-transfer process, *para*-bromine of BDE-47 is preferentially debrominated and generate BDE-17. The debromination reactivity of these bimetallic systems at 1% (w/w) metal additive loading follows as: Fe/Pd > Fe/Ag > Fe/Cu > Fe/Ni > Fe/Au > Fe/Pt ≈ nZVI (Fig. 10C and D).

#### 2.2.2. Defluorination

**Fig. 11:**
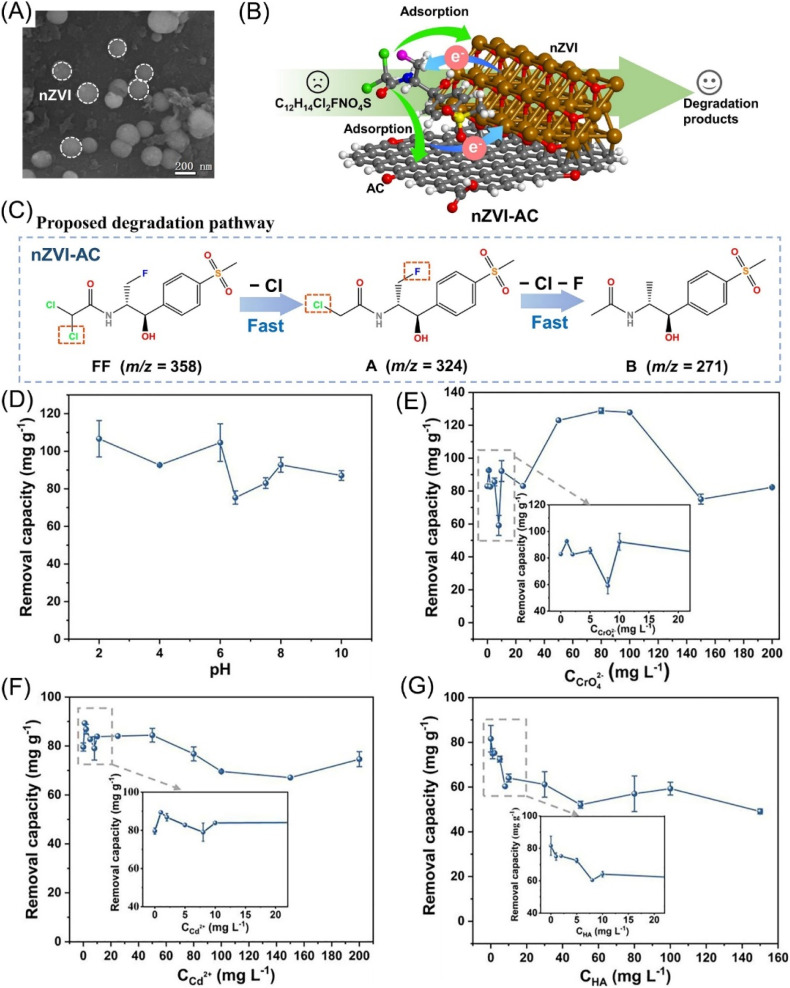
(A) SEM morphology of nZVI; (B) scheme of the adsorption and dehalogenation of FF by nZVI-AC; (C) proposed degradation pathway of FF by nZVI-AC; removal capacity of FF by nZVI-AC as affected by (D) initial pH, (E) CrO_4_^2−^, (F) Cd^2+^, (G) HA,^94^ copyright 2024 Elsevier.

As the chemical reactivity of C–F is significantly lower than C–Cl and C–Br, research on defluorination by nZVI is insufficient.^94^ Huang *et al.* used activated carbon-supported nZVI (Fig. 11A) to remove florfenicol (FF), which is a widely used halogenated antibiotic.^95^ The removal of FF was demonstrated as adsorption and dehalogenation, the former of which is dominated by van der Waals forces, hydrogen bonding, and chemisorption (*e.g.*, π–π interaction and Fe–O bond). The adsorbed FF was subsequently suffered from dechlorination and defluorination by nZVI-AC, and generated no harmful products (Fig. 11B and C). The removal of FF performed a pH and Cr(vi) concentration-dependent response (Fig. 11D and E), and Cd(ii) slightly affected the removal efficiency (Fig. 11F). Acidic conditions would facilitate the formation of H_2_ and prevent the passivation of nZVI by providing abundant H^+^ ions, and thus promote the removal of FF. The redox reaction of Cr(vi) with nZVI and Fe(ii) may enhance the electron transfer and hence promote the removal of FF by nZVI. However, Cr(vi) may compete with FF for reactive sites in the condition of high concentration of Cr(vi). As regard to the coexisting Cd(ii), it could not only increase the ionic strength and restrain the iron oxide precipitation and enhance the FF removal, but also could compete with FF for reactive sites and inhibit the removal of FF, resulting in a slight impact on the FF removal on the whole. While humic acid has obvious suppression on the removal of FF (Fig. 11G), due to the competitive adsorption of humic acid (HA) on the shells of nZVI by chelating.

### 2.3. Reduction of metal/metalloid ions, nitrate, and nitro

Metal/metalloid ions, such as Cr(vi), Pb(ii), and Sb(v), which are widely used in many fields, have become common toxic pollutants due to improper storage or uncontrolled emission.^56^ They can exist in the environment for a long time and get into human bodies *via* inhalation and contact, posing an increasing risk of disease and cancer for human beings.^56,62^ The extensive use of chemical fertilizers and nitrate-containing materials has accelerated the excessive accumulation of nitrate in water bodies, resulting in hydration and red tide in eutrophicated water.^76,96^ Nitro compounds, which are widely utilized in the production of insecticides, explosives, and dyes, have become one of the major organic pollutants in water owing to their degradation-resistant characteristics, carcinogenicity, and teratogenicity on organisms.^54^

In recent years, extensive research efforts have been dedicated to endowing nZVI materials with multiple functions and expanding the applications of nZVI towards these pollutants, leading to the development of various nZVI-based composites that not only preserve their reductive potential but also outperform their counterparts in the recovery of pollutants from environment.

#### 2.3.1. Direct reduction

Highly toxic Cr(vi) can be reduced to less toxic Cr(iii) by nZVI (eqn (2) and (3)). Hao *et al.* prepared nZVI by a low-cost green synthetic method using plant extracts for the reduction of Cr(vi).^97^ These biomolecules can cap on the Fe^0^ surface and prevent the oxidation and inactivation of nZVI. Similarly, Cheng *et al.* adopted CaCO_3_ coated on nZVI for the reduction of Cr(vi), which performed long-term stability.^98^ Their study demonstrated that the presence of Ca^2+^ and Mg^2+^ facilitated the reduction of Cr(vi), but the PO_4_^3+^ and HA impeded the reduction.

Although nitrate reduction by nZVI-based materials has been extensively studied, the aggregation and surface passivation of nZVI, interference of coexisting ions, and the preference of unfavored ammonia products limit the application of this technology. Huang *et al.* supported nZVI/Pd on graphene to overcome the aggregation of nZVI and the extensive formation of ammonia, in which the N_2_ selectivity was enhanced from 0.4% to 15.6% and the nitrate removal efficiency was 97%.^99^ Polymers, including polyacrylamide (PAA), carboxymethyl cellulose (CMC), polyethylene sorbitan monolaurate (PSM) and polyvinylpyrrolidone (PVP) have also been employed for the inhibition the aggregation of nZVI during the reduction of nitrate, in which the reduction efficiency followed the order as 99.5% (PVP-nZVI) > 99% (PAA-nZVI) > 97% (PSM-nZVI) > 70% (CMC-nZVI) > 55.6% (pristine nZVI).^75^ Moreover, scholars discovered that doped metal (Ni, Ag, Cu) can promote electron transfer and minimize oxidation of nZVI, and alkaline conditions are favorable for the removal of nitrate.^74^ Some optimization experiments indicated that the main effects that impact the reduction efficiency of nitrate followed the order as: aging time > pH > temperature.^76^

**Fig. 12:**
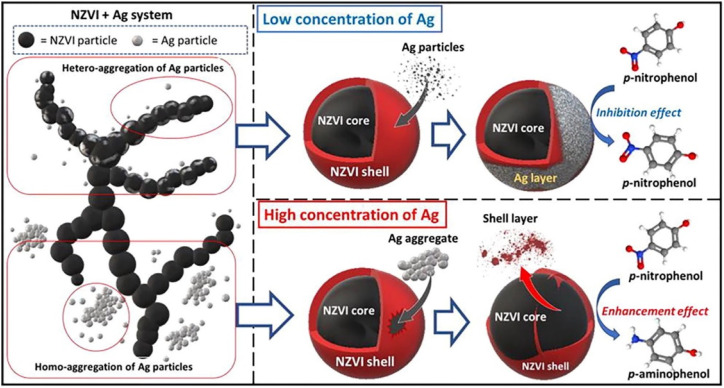
The effect of Ag loading on the reduction of *p*-NP by nZVI,^52^ copyright 2022 Elsevier.

Nitro compounds can also be primarily reduced to amino compounds, which are more prone to be bio-degraded.^54^ Gu *et al.* employed graphene/biochar in the supporting of nZVI to enhance the electron-releasing capacity and the electron transfer from nZVI or Fe(ii) to –NO_2_, achieving a removal efficiency of 77.1% towards nitrobenzene.^54^ Deng *et al.* demonstrated a high dose of Ag on nZVI will not only prevent the aggregation of nZVI, but also facilitate the nZVI peeling, thus benefit for the reduction of *p*-nitrophenol; while, low dose of Ag on nZVI has an adverse effect (Fig. 12).^52^

Based on its high reductivity, investigations of reductive recovery of Cu and Ni from polluted rivers, reductive degradation of organophosphate esters, and reductive degradation of sulfamethoxazole by nZVI have also been reported.^45,68^

#### 2.3.2. Reduction–precipitation

In the reduction of Cr(vi) by nZVI, the generated Cr(iii) can form co-precipitate with Fe(ii) and Fe(iii) (eqn (4)), which can significantly alleviate the bioavailability of Cr in water of soil.^57^ Rice straw-derived hydrothermal carbon and cellulose filter paper have been used as support for nZVI, aiming to improve the dispersibility and oxidation resistance of nZVI during the reduction of Cr(vi).^100,101^

**Fig. 13:**
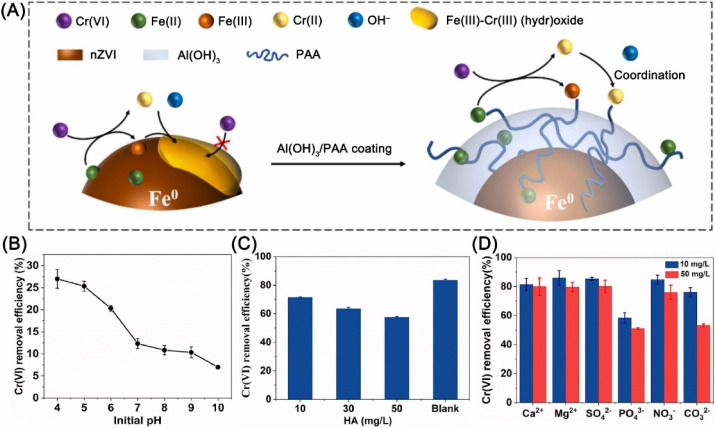
(A) Illustrations of the *in situ* anti-passivation functions in the reduction–precipitation of Cr(vi) by PAA/Al(OH)_3_ coating nZVI,^58^ copyright 2021 Elsevier. Effects of (B) initial pH, (C) HA concentration, and (D) coexisting inorganic ions on Cr(vi) removal efficiency by BC/nZVI,^57^ copyright 2021 Elsevier.

Most of the Cr can be recovered from water utilizing the magnetically separating of nZVI material. While the cover of precipitate may reduce the activity of nZVI. Therefore, Liu *et al.* employed nZVI supported by xanthan gum-modified reduced graphene oxide for the remediation of Cr(vi)-polluted aquifer, in which Cr(vi) is reduced to Cr(iii) by Fe^0^ and then adsorbed by the negatively charged oxygen-containing functional groups on rGO and formed precipitate attached to rGO, rather than on the surface of nZVI.^56^ While, Hu *et al.* confined PAA on the surface of nZVI by Al(OH)_3_, the results of which showed that the surface carboxylic groups of PAA bound generated Cr(iii) and Fe(iii) and suppressed the precipitation of hydroxides on the surface of nZVI (Fig. 13A).^58^ Thus, Cr(vi) reduction capacity was improved from 49.4 to 92.6 mg g^−1^ within 24 h. A study conducted by Liu *et al.* confirmed that alkaline conditions are unfavorable for the reduction of Cr(vi) (Fig. 13B) due to the rapid precipitation of iron ions on the surface of nZVI, preventing the reaction of Fe^0^ with Cr(vi).^57^ In addition, Ca^2+^, Mg^2+^, SO_4_^2−^, and NO_3_^−^ have a slightly effect on the removal of Cr(vi), while HA, PO_4_^3−^, and high concentrations of CO_3_^2−^ can reduce Cr(vi) removal efficiency (Fig. 13C and D).

Besides Cr(vi), Fan *et al.* fabricated hydrotalcite-supported nZVI for the removal of methylene blue (MB).^50^ MB is firstly reduced to colorless leuco-MB, which is simultaneously attached to the surface of nZVI and finally removed by complexation precipitation.

2 Cr_2_O_7_^2−^ + 2Fe^0^ + 7H_2_O → 2Fe^3+^ + 2Cr^3+^ + 14OH^−^

3 Cr_2_O_7_^2−^ + 6Fe^2+^ + 7H_2_O → 6Fe^3+^ + 2Cr^3+^ + 14OH^−^

4 *x*Cr^3+^ + (1 − *x*)Fe^3+^ + 3OH^−^ → Cr_*x*_Fe_1−*x*_(OH)_3_↓

#### 2.3.3. Adsorption–reduction

Adsorption is identified as the most reliable method for the enrichment of pollutants with low concentration or high mobility, which will promote the following remediation process undoubtedly.

**Fig. 14:**
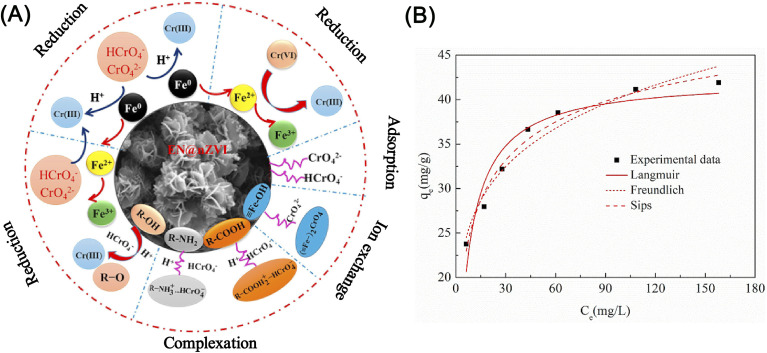
(A) Illustrations of the possible reaction mechanism of Cr(vi) removal by EN@nZVI; (B) isotherm for adsorption of Cr(vi) on EN@nZVI,^102^ copyright 2020 Elsevier.

Li *et al.* developed a surface phosphate modification for nZVI to enhance the adsorption of Cr(vi) by revising the monodentate mononuclear model on pristine nZVI into bidentate binuclear one on the phosphate nZVI surface, thus promoting a removal efficiency of Cr(vi) by 4 folds.^60^ Using ammonium thiocyanate functionalized graphene oxide supported nZVI, Wang *et al.* found that the adsorption process follows as pseudo-second-order model and Langmuir–Hinshelwood first-order model at the reduction process, and the removal of Cr(vi) was dominated by chemical surface-limiting step.^103^ In the study conducted by Shu *et al.*, they identified that the adsorption–reduction of Cr(vi) by almond shell-derived biochar/nZVI prefers acidic conditions rather than alkaline conditions (eqn (5) and (6)).^104^ The results indicated that N–H was the main functional group responsible for the chemisorption process, and the removal efficiency of Cr(vi) was 99.8% at pH 2–6, which was approximately 20% higher than that at pH 7–11. Other functional groups, *i.e.* R-COOH, R-OH, R-NH_2_, and R-C-O-C on nZVI materials could also provide active sites during the chemisorption process, which can be described by Sips adsorption isotherm model and pseudo-second order model as well, involving adsorption, surface complexation, reduction and ion exchange reaction (Fig. 14).^102^

5 HCrO_4_^−^ + Fe^0^ + 7H^+^ → Fe^3+^ + Cr^3+^ + 4H_2_O

6 Cr_2_O_7_^2−^ + 2Fe^0^ + 14H^+^ → 2Fe^3+^ + 2Cr^3+^ + 4H_2_O

**Fig. 15:**
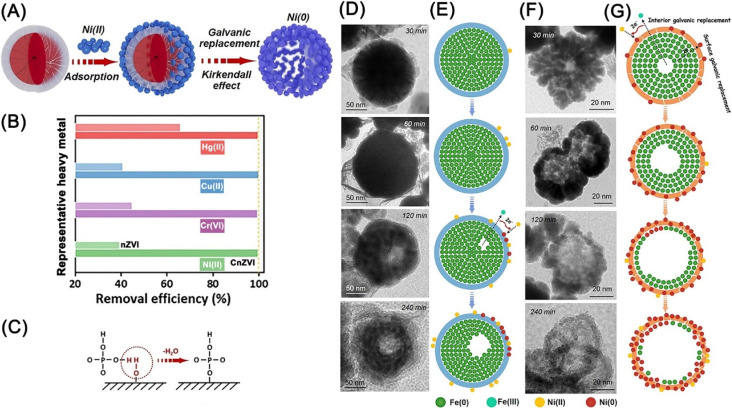
(A) Illustration of the immobilization of Ni(ii) by CnZVI; (B) removal efficiencies of Ni(ii), Cr(vi), Cu(ii), and Hg(ii) with nZVI and CnZVI; (C) illustration of the adsorption of HPO_4_^2−^ on the surface of nZVI; (D) time-sequence void evolution within iron spheres during Ni(ii) removal with nZVI; (E) illustration of the Ni(ii) removal process in a single sphere of nZVI; (F) time-sequence void evolution within iron spheres during Ni(ii) removal with CnZVI; (G) illustration of the Ni(ii) removal process in a single sphere of CnZVI,^63^ copyright 2021 Wiley.

Sb(v) have aroused worldwide concern owing to its high toxicity and broad application, and the reductive transformation of soluble Sb(v) to more easily precipitable Sb(iii) compound (Sb(OH)_3_) is a promising approach to remediating Sb(v)-contaminated water.^62^ As the main form of Sb(v) is Sb(OH)_6_^−^, Zhou *et al.* introduced microbial extracellular polymeric substances on nZVI to decrease the electrostatic repulsion between nZVI and Sb(v), thus strengthening the adsorption and reducibility of Sb(v) by nZVI at a maximum removal capacity of 202 mg g^−1^.^62^ Song *et al.* applied nanocelluloses affixed nZVI for the adsorption of Ni(ii) *via* physical adsorption at the interface and reduction of Ni(ii), achieving an enrichment and recovery of 99.5% Ni(ii).^64^ Li *et al.* used phosphorylated nZVI for the removal of metals/metalloids (Ni(ii), Cu(ii), Cr(vi), and Hg(ii)) *via* modulated adsorption and boosted Kirkendall effect (Fig. 15A and B).^63^ The adsorption of positively charged metal ions is promoted by surface electronegativity deriving by HPO_4_^2−^, which is formed through a typical dehydration process of dihydrogen phosphate groups (Fig. 15C). Meanwhile, the radial nanotracks (CnZVI), generated from the ensile hoop stress on nZVI by phosphorylation, promote the facile inward diffusion of Ni(ii) and the rapid outward transfer of electron and Fe(ii) through oxide layer, namely galvanic replacement (Fig. 15D–G).

Han *et al.* discovered that boric acid and borates can be transformed as B–B/B–Fe on nZVI, providing highly electron-deficient Lewis's acid sites for the effective gathering of NO_3_^−^ and OH^−^, leading to a high-efficiency adsorption–reduction of nitrate in a wide range of pH (5–9).^73^ Yang *et al.* entrapped nZVI in the matrix of cyclodextrin polymer for the adsorption and reduction of *p*-nitrophenol, and the material had a long-term stability of 109 d.^51^ Surfactants, such as rhamnolipid and sodium dodecyl sulfate have also been applied in the strengthening of the adsorption and reduction of nitrobenzene by nZVI from soil.^53^

Adsorption and decontamination of hydrophobic BDE-3 from aqueous solutions is commonly difficult. Sophorolipid-modified nZVI, which possessed more accessible active sites and reduced charge transfer resistance compared to pristine nZVI, enhanced the solubilization of BDE-3 *via* halogen bonding interaction, giving significant facilitation on the adsorption and subsequent reduction by nZVI and achieving a removal efficiency of 99.96%.^55^

When the composition of contaminants is complex, competitive adsorption or reaction may occur. Cai *et al.* identified that Pb(ii) and Ni(ii) will compete the limited effective adsorption/reaction sites in binary mixtures, and Pb(ii) owns greater competitive ability than Ni(ii).^65^ Liu *et al.* modified nZVI/rGO with xanthan gum to fabricate a stable reaction zone, for the breaking of the negative synergistic phenomenon between NO_3_^−^ and Cr(vi).^105^

#### 2.3.4. Adsorption–reduction–precipitation

The efficient removal of non-biodegradable metals/metalloids has been one of the top priorities in wastewater remediation, thus making the development of corresponding green technologies of great significance.^63^ In recent years, extensive research efforts have been dedicated to enhancing the removal of metal/metalloid ions by the method of adsorption–reduction–precipitation, leading to the development of novel nZVI magnetically separable composites that make the thorough removal of metals/metalloids or recovery of resources from wastewater achievable. Continuous and inexhaustible efforts have been devoted to the exploration of nZVI material with high and selective adsorption capacity, high and sustained reductivity, and high separability.

**Fig. 16:**
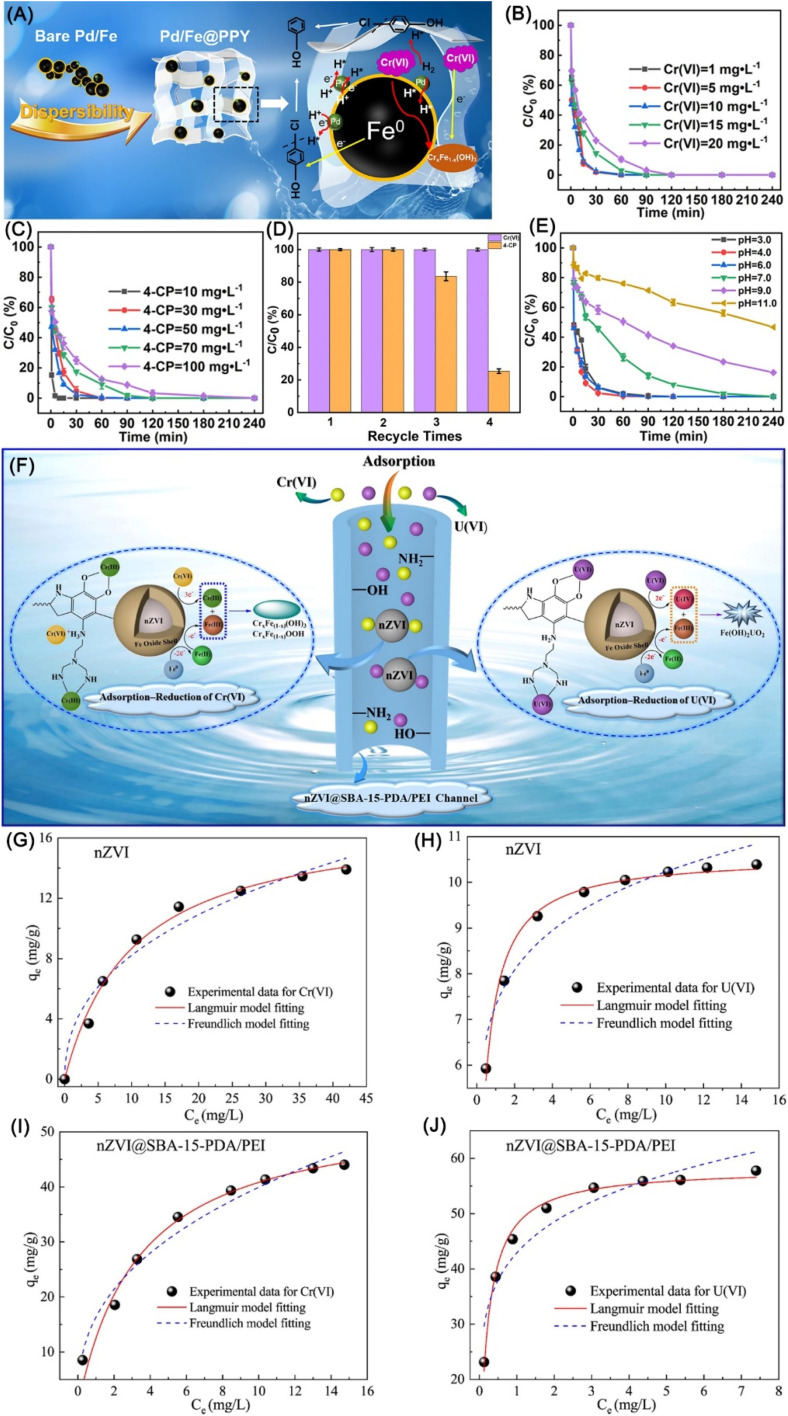
(A) Mechanistic illustration for simultaneous removal of 4-CP and Cr(vi) by Pd/Fe@PPY; effects of initial (B) Cr(vi) and (C) 4-CP concentration on the removal efficiency, (D) recyclability of Pd/Fe@PPY in the removal of 4-CP and Cr(vi), (E) effects of initial solution pH on the removal efficiency,^126^ copyright 2022 Elsevier. (F) Schematic diagram of the possible adsorption–reduction–precipitation mechanism for Cr(vi) and U(vi); (G–J) sorption isotherms of Cr(vi) and U(vi) fitted by the isotherms equations,^71^ copyright 2021 Elsevier.

Converting highly toxic Cr(vi) to low-toxic and separable immobilized Cr(iii) by nZVI *via* adsorption–reduction–precipitation has garnered increasing research attention. As there are various limitations of pristine nZVI during production and application, including easy agglomeration, fast oxidation, and easy deactivation,^61,106^ nZVI is commonly loaded in porous materials, such as bentonite,^107^ attapulgite,^108^ SBA-15,^109^ graphene,^110^ biochar,^111–116^ resin,^117^ hydrogel,^118–120^ membrane,^121,122^ MOF^106^*etc.* The supporting not only prevents aggregation and deactivation of nZVI, but also endows nZVI materials with high and/or selective adsorption towards Cr(vi). Xu *et al.* implemented a series of spectroscopic investigations and verified that various oxygen-bearing functional groups on biochar benefit the adsorption of Cr(vi) *via* complexation and electrostatic interaction.^113^ The protonation of –NH_2_ and –OH on support can also significantly enhance the adsorption of Cr(vi) *via* electrostatic attraction, leading to a dominant chemisorption mechanism following a pseudo-second order model.^108,116^ In addition to the contribution to adsorption, carbon and GO can also provide good electrical conductivity and long-term electron-releasing properties, accelerating the reduction of Cr(vi) to Cr(iii).^110^ Moreover, the reactivity of supported nZVI can be further improved by modifying supported nZVI with stabilizers, such as soluble starch, polydopamine, and glutathione.^108,111,121,123^ Besides adsorption and reactivity of nZVI, the removal efficiency of Cr(vi) is also subjected to material status, interfere ions, pH, and catalyst. Normally, nZVI materials in suspension (without drying process) performed higher removal efficiency compared with powder ones, acidic and anaerobic conditions facilitate the reaction.^123,124^ Acetate ions could promote the Cr(vi) removal, but HA, nitrate, and carbonate ions resulted in negative effects, while SO_4_^2−^ and Cl^−^ have slight effect on the removal efficiency.^61,109^ The removal of Cr(vi) can be hugely enhanced by the catalyzing of Pd or Ni, which catalyze the formation of H· and benefit for the rapid reduction of Cr(vi) (eqn (7) and (8)), leading to a complete reduction of Cr(vi) in 5 min.^109,125^ All in all, the removal of Cr(vi) by nZVI involved three steps, (i) adsorption of Cr(vi) by active sites, (ii) reduction of Cr(vi), and (iii) formation of Fe–Cr–O precipitates; the adsorption accounts for ∼30% on the removal of Cr(vi), the reduction/precipitation accounts for the rest ∼70%.^108,118,125^ Lei *et al.* reported a simultaneous adsorption–reduction–precipitation removal of Cr(vi) and 4-NP by polypyrrole-supported Pd/nZVI (Pd/Fe@PPY), which achieved complete removal of Cr(vi) and 4-CP within 1 min and 60 min respectively (Fig. 16A–C).^126^ The presence of Cr(vi) significantly impaired the dechlorination of 4-CP attributing to the deposition of Cr(iii)–Fe(iii) co-precipitates on nZVI, while the presence of 4-CP had a minor effect on the removal of Cr(vi) and total Cr, which is more obvious in recycle experiments (Fig. 16D). Similarly, the combined removal of Cr(vi) and 4-CP prefers acid conditions rather than alkaline conditions (Fig. 16E).7
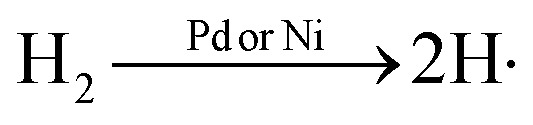

8 HCrO_4_^−^ + 3H· + 4H^+^ → Cr^3+^ + 4H_2_O

U(vi) and U(iv) are two dominating states of Uranium, the former of which is higher in mobility and solubility in aquatic environments, lower bioavailability, and more chemical toxicity.^70,127^ Therefore, the removal and transformation of U(vi) from contaminated environment has been an urgent matter to be solved. Scholars confirmed that the reduction of U(vi) by nZVI could easily form precipitates and thus eliminate their immobilization in the natural environment (eqn (9) and (10)). Zhang *et al.* employed nZVI loaded chitosan in the synergistic adsorption (Langmuir isotherm adsorption) and reduction of U(vi), achieving a high removal quantity of 591.72 mg g^−1^.^70^ The well-dispersed nZVI owed high reactivity and is considered as electron donor in the chemical reduction of mobile U(vi) to precipitated U(iv) on nZVI. Li *et al.* developed an adsorption–reduction–solidification strategy for the elimination of U(vi) by MCM zeolite-supported nZVI, yielding a U(vi) removal capacity on nZVI/MCM of 216 mg g^−1^.^127^ The surface functional groups, including –OH, Fe–O, and Si–O, are beneficial for the adsorption of U(vi). Hua *et al.* explored the technique of reduction, enrichment, and separation of U from U-tailings wastewater in a continuous-flow device, decreasing the concentration of U from 331 μg L^−1^ to 1.47 μg L^−1^ in the continuously dealing of ∼500 L radioactive wastewater in 193 h.^72^

Liu *et al.* presented a novel collaborative strategy for enhancing the removal of U(vi)/Cr(vi) by MBenes/SBA-15 supported nZVI, in which U(vi)/Cr(vi) were rapidly and abundantly adsorbed *via* electrostatic interaction and reduced by surface-associated nZVI to U(iv)/Cr(iii), and finally, the generated U(iv)/Cr(iii)/Fe(iii) rapidly formed co-precipitation on the nZVI composite's surface (Fig. 16F–J).^23,71^ The competitive adsorption of U(vi) and Cr(vi) can occur in an insufficient adsorption sites situation, which may result in low removal efficiency.^71^

9 UO_2_^2+^ + Fe^0^ → UO_2_↓ + Fe^2+^ + Fe^3+^

10 UO_2_^2+^ + 2Fe^2+^ → UO_2_↓ + 2Fe^3+^

The adsorption–reduction–precipitation method has also been applied in the recovery of As(v), Ni(ii), and Pb(ii). Fan *et al.* adopted nZVI/biochar in the reduction of As(v) to As(iii), which was subsequently absorbed by amorphous FeOOH and co-precipitated on nZVI.^69^ Sang *et al.* demonstrated the formation of NiO, Ni(OH)_2_, and Ni during the recovery of Ni(ii) by rhamnolipids modified nZVI, suggesting a reduction–adsorption–precipitation mechanism involving in ref. 66. Similarly, precipitate PbO, Pb(OH)_2_, Pd_3_(CO_3_)_2_(OH)_2_, and Pb^0^ can be discovered on nZVI during the remediation process, revealing the synergistic effects of adsorption, reduction, and precipitation.^67^

### 2.4. Concept for future

When employing nZVI in the reduction of contaminants, its reducing activity and long-term stability are significant for the reaching of its full potential in practical application. Although nZVI demonstrated superior reduction of numerous organic contaminants, metal ions, and nitrate from wastewater, weak van der Waals forces and intrinsic magnetic interactions led to strong homo-aggregation of nZVI, posing unavoidable challenges to its reactivity and long-term performance. Meanwhile, nZVI with high reducing activity is vulnerable to faster dissolution and surface passivation, also giving drawbacks to its long-term stability. Besides, nZVI with low reducing activity is incompetent in providing sufficient reductivity for contaminants. Therefore, high-activity cultivation and maintenance technologies and surface passivation protection technologies, which can prevent the inactivation of surface-active sites of nZVI and sustain its high activity during application, may be a necessity. The present solution is loading nZVI in/on porous materials to disperse nZVI and prevent their aggregation, alleviate their oxidation and deactivation, and further endow nZVI materials with higher adsorption capacity or cooperative remediation effects. Future efforts may focus on the further improvement of reductivity and long-term stability of nZVI to deal with refractory contaminants and broaden their reductive application, as well as the electrochemical properties of support. Such as expanding the application of nZVI in dehalogenation, *i.e.* debromination and defluorination, to deal with the increasingly severe organic fluorine and organic bromine pollutions.^128,129^

Future studies may also develop nZVI materials with high/specific affinity towards contaminants by regulating the surficial characteristics, devoting to the remediation of actual wastewater with low concentration. Considering that the components of actual wastewater are always complex, investigations and deeper view on the interactions and mechanisms during the remediation process also need to be clarified.

As for the comprehensive utilization of nZVI, the dissolving Fe(ii) during dechlorination may have a secondary utilization value, as a study has shown that Pd/Fe(OH)_2_ has reductive dechlorination on TCE.^32^ Thus, utilization protocols of dissolved Fe(ii) generated from the corrosion of nZVI and the mechanisms involved in are deserve to be explored.

## 3. Electrolysis-assisted reduction by nZVI

Recently, an electro-remediation technology using ZVI has been proposed for the remediation of metal ions, organic pollutants, and nitrate. With the additional electrons provided by the electric current, the reduction of contaminants by nZVI can be enhanced.^130^

**Fig. 17:**
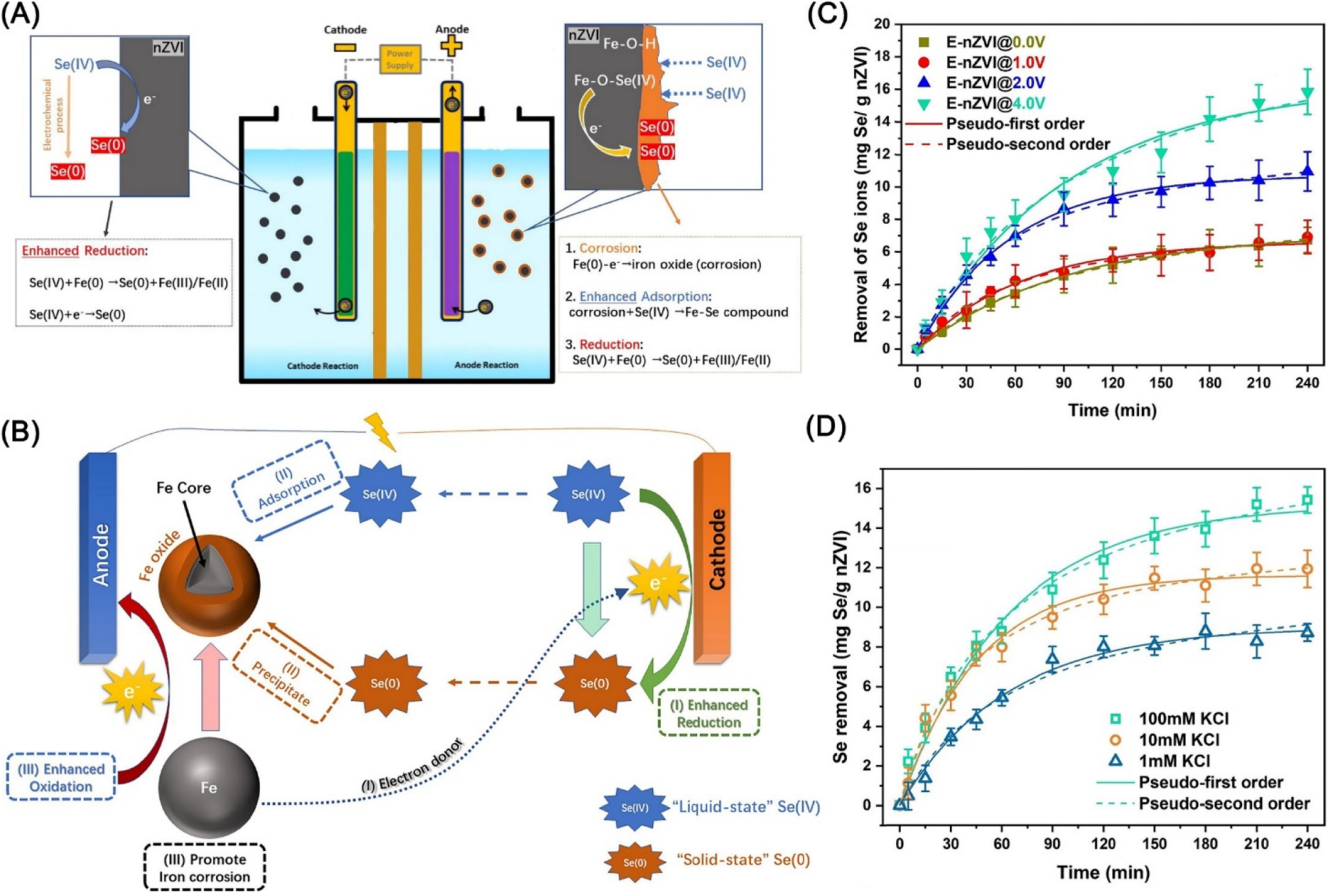
(A) Schematic diagram of the electrolysis-assisted nZVI system for the removal of selenite; (B) schematic diagram for proposed mechanisms of the removal of selenite; Se(iv) removal kinetics by the E-nZVI system at different (C) applied voltages and (D) electrolyte concentration,^131^ copyright 2021 Elsevier.

Qi *et al.* proposed an efficient technique to enhance the removal of Se(iv) from wastewater *via* the electrolysis-assisted reduction by nZVI (E-nZVI), which achieved an enhanced removal efficiency (15.83 mg Se(iv)/g nZVI) exceeded nZVI system by ∼135%.^131^ Separate anode–cathode experiments and surface-sensitive quantitative characterization demonstrated that improved performance is not only attributed to the synergistic effects of nZVI and cathodic reduction on the precipitation of Se, but also benefit from the nZVI corrosion aggravated by electrochemical oxidization in the anode chamber (Fig. 17). Kinetic tests indicated that lowering the resistance (elevating electrolyte concentration) and raising the applied voltage (4.0 V or more) can give rise to promotion in the removal efficiency (Fig. 17), but cannot give rise to apparent promotion on the reaction rate constant.

Pavelková *et al.* conducted dechlorination tests of chlorinated hydrocarbons (TCE, PCE, and DCE) by nZVI with the electric field and indicated that the dechlorination predominantly occurred around the anode despite that it is expected near the cathode.^130^ No dechlorination products was detected in the cathode owing to the presence of Fe oxides, such as Fe^3+^ or Fe(OH)_4_^−^. The electrode reaction produces abundant H^+^, which can prevent the deactivation of nZVI and enhance the dechlorination performance (eqn (11)).

11 R-Cl + H^+^ + Fe^0^ → R-H + Cl^−^ + Fe^2+^

**Fig. 18:**
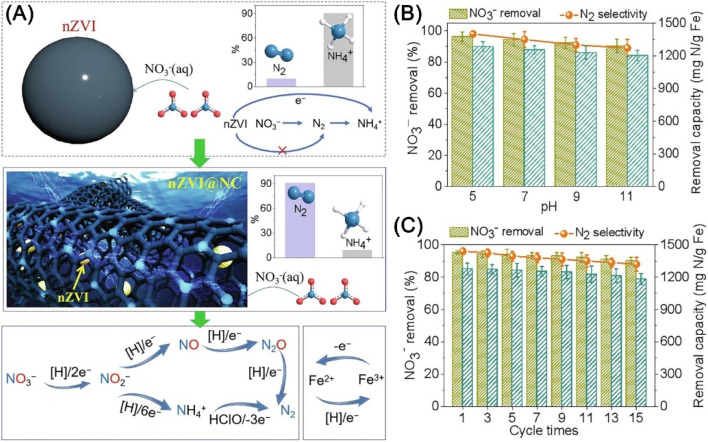
(A) Mechanisms of catalytic denitrification on the nZVI@NC; the effect of (B) pH and (C) cycle times on nitrate removal and N_2_ selectivity with nZVI@NC,^133^ copyright 2021 Elsevier.

There are also reports on the enhancement of nitrate removal and nitrogen selectivity by electrochemical method with magnetically immobilized nZVI anode on RuO_2_–IrO_2_/Ti plate, which possesses with ammonia-oxidizing function.^132^ The electrochemical method showed a nitrate removal efficiency of 94.6% and nitrogen selectivity of 72.8% at pH 3.0, and nitrate removal efficiency of 90.2% and nitrogen selectivity of 70.6% near a neutral medium (pH = 6). Wang *et al.* adopted tubular nitride carbon encapsulated nZVI as an electrocatalyst for the regulating of multi-electron transfer reaction, which exhibited superior nitrate removal efficiency (92%) and N_2_ selectivity (97%) in the pH range of 5–11 and recyclability (Fig. 18).^133^ The superior performance is attributed to the selectivity adsorbing of nitrate on the porous and hydrophilic nitride carbon layer, and the subsequent efficient cleavage of N–O bond by gaining electrons from nZVI and activated H. In addition, the generated intermediate NH_4_^+^ can be oxidized to N_2_ by HClO from the anode, thus leading to the high promotion of the production of N_2_ (Fig. 18).

**Fig. 19:**
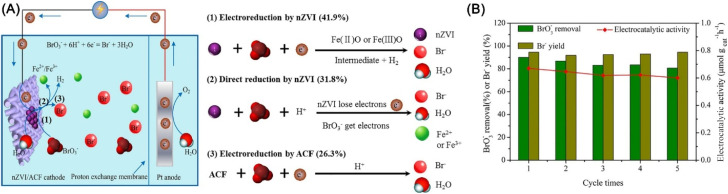
(A) Mechanism of electrocatalytic BrO_3_^−^ reduction by nZVI/ACF; (B) electrocatalytic reduction performance of BrO_3_^−^ during five cycles,^134^ copyright 2020 Elsevier.

Bromate (BrO_3_^−^) is a disinfection by-product originating from the ozonation or chlorination of Br^−^ in drinking water sources.^134^ Yao *et al.* used nZVI immobilized activated carbon fiber electrode for the electrocatalytic reduction of BrO_3_^−^, achieving a reduction efficiency of 94.2% of BrO_3_^−^ (1.22 μM) within 90 min.^134^ In the accomplishment of BrO_3_^−^ reduction, direct reduction by nZVI, electrocatalytic reduction by nZVI, and electrocatalytic reduction by activated carbon fiber account for 31.8%, 41.9%, and 26.3%, respectively (Fig. 19). Recycle tests indicated this method has excellent performance during successive reduction. The reduction favors low dissolved oxygen concentration and pH, and high current intensity (Fig. 19).

### 3.1. Concepts for the future

Future research employing this innovative remedial technology may focus on the potential scale-up application at a contaminated site and the decontamination efficiency, or the reduction degradation of recalcitrant contaminants with the aid of a catalyst. The factors that affect the contaminant removal efficiency and energy consumption, in terms of corrosion voltage, corrosion current density, charge-transfer resistance, and the modification of nZVI on electrode, also deserves to be explicated. Furthermore, the strategy that realizing a circulation of formation, remediation, and regeneration of high reactivity nZVI in electrolysis-assisted application is also worth to be attempted.

## 4. Bacterium-assisted reduction by nZVI

Currently, the coupling treatment method of nZVI with microorganisms is a research hotspot in the field of organic degradation, nitrate reduction, and metal ion removal.^135^

**Fig. 20:**
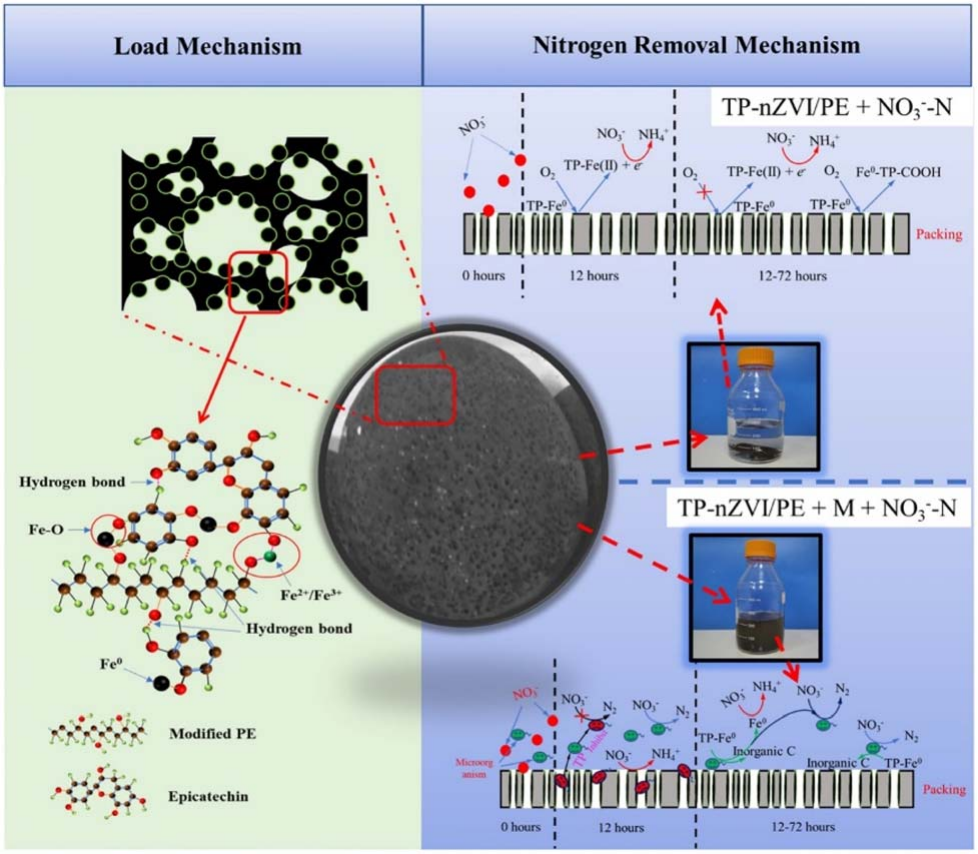
Schematic diagram of the highly efficient and selective transformation of nitrate to N_2_ with the assistance of microorganisms and TP-NZVI/PE,^136^ copyright 2020 Elsevier.

Ma *et al.* employed a coupling system of nZVI and microorganisms to degrade polybrominated diphenyl ethers, and achieved complete debromination of BDE-209 within 30 days (Fig. 20).^137^ The addition of nZVI in sediment microbial fuel can reduce the oxidation–reduction potential of the system and thus enrich dechlorination microbial communities beneficial for the reductive degradation of PCBs.^138^ The combination of nZVI with microorganisms has also been applied in the bio-nano-dechlorination of TCE, bisphenol A, bisphenol S, pentachlorophenol, dichloromethane and 1,2-dichloroethane.^139–143^ The major removal improvement mechanisms involve chemical reduction by nZVI and dehalogenation bacterium-mediated microbial dissimilatory iron reduction (*Longilinea* and *Desulfofustis*, *Dechloromonas* sp., *Shewanella putrefaciens* CN32, *Terrimonas*, *Lysobacter*, *Acidovorax*, *Dehalococcoides mccartyi*, and *Burkholderia ambifaria* strain L3).^138,139,141,142,144,145^ Xu *et al.* proposed a removal mechanism of organic halides as: (i) organohalide-respiring bacteria utilize H_2_ generating from nZVI corrosion to enhance dechlorination in the short term; (ii) the adsorption of nZVI materials and the promotion of dechlorination by attached biofilm in long-term.^146^ Li *et al.* suggested that the addition of nZVI can significantly increase the dehydrogenase activity of indigenous microorganisms in soil, and thus promote the removal of organochlorine pesticides.^147^

The addition of nZVI in anaerobic/anoxic/aerobic membrane bio-reactor can provide electrons for the denitrification, thus enhancing the reduction of nitrate.^148^ Similarly, Zhang *et al.* adopted biochar-supported nZVI in the cathodic removal of nitrated in a bioelectrochemical system to enhance the reduction of nitrate to NH_4_^+^.^149^ While the environmental-friendly reductant of nitrate is N_2_. It is worth mentioning that Zhou *et al.* used tea polyphenols to realize an excellent distribution and anti-oxidization of nZVI, achieving high conversion and selectivity in the transformation of nitrate to N_2_ with the assistance of microorganisms (Fig. 20).^96,136^

Wang *et al.* developed a novel porous phosphate-solubilizing bacteria beads loaded with biochar/nZVI in the enhancement of the passivation of lead in soil.^150^ nZVI makes the soil environment to be a relatively reduced state and thus promotes the release of soluble phosphate from soil, which can further form insoluble precipitates with Pd(ii), transforming Pb(ii) to a stable fraction alone in company with the reduction of Pb(ii) by nZVI.

**Fig. 21:**
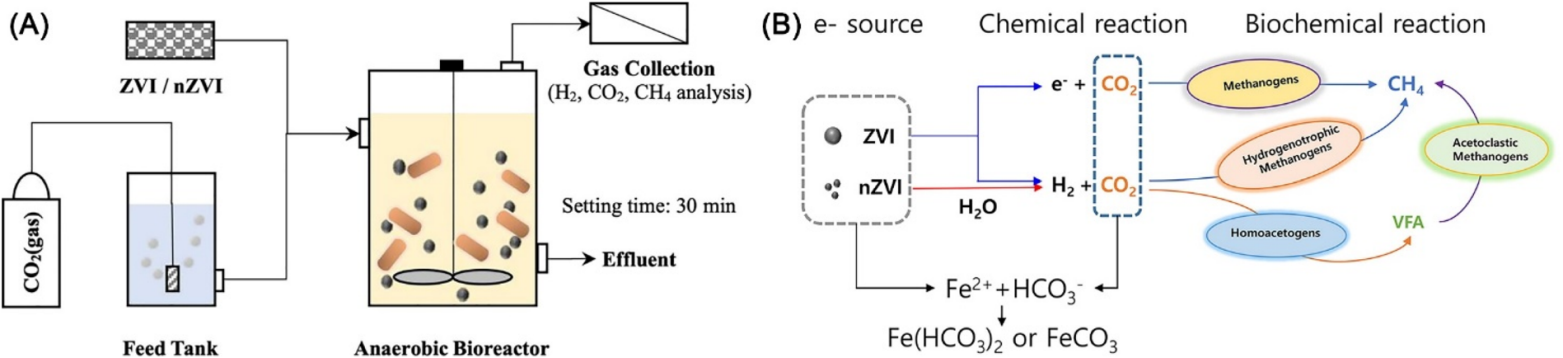
(A) Experimental setup of anaerobic bioreactor for CO_2_ biomethanation with continuous addition ZVI and nZVI; (B) schematic process of CO_2_ biomethanation with ZVI and nZVI,^151^ copyright 2022 Elsevier.

As CO_2_ is one of the major greenhouse gases causing global climate change, its reduction has become a key global issue. nZVI is found to have the potential for biomethanation of CO_2_ under anaerobic conditions in the bioreactor (Fig. 21), which relies on the formation of H_2_ for hydrogenotrophic methanogenesis by nZVI (eqn (12)–(14)).^151^ When nZVI is continuously added, 11.52% of the dissolved input CO_2_ is transformed into CH_4_. While, the economic evaluation of this operation remains to be investigated, for the reason that the production of nZVI also required NaBH_4_, which is actually a good source of hydrogen.

12 2H_2_O + Fe → H_2_ + Fe^2+^ + 2OH^−^

13 CO_2_ + 4H_2_ → CH_4_ + 2H_2_O

14 CO_2_ + 4Fe + 8H^+^ → CH_4_ + 4Fe^2+^ + 2H_2_O

### 4.1. Concepts for the future

Future research is suggested to focus on the diverse dehalogenase genes that are essential for microbial dechlorination, debromination, or denitrification, optimize the coupling system with different modified or doped nZVI, explore novel collaborative remediation methods involving multiple functional microorganisms with dehalogenase genes achieved by genetic engineering, and consider the influence of field environmental factors on the removal efficiency. Furthermore, the evolution mechanism of added nZVI in microbial-assist remediation warrants further exploration.

## 5. Conclusions

nZVI possesses high reactivity towards various contaminants and is attracting increasing attention. In the pursuit of superior and sustained reductivity of nZVI during application, many research has been conducted on the development of nZVI technology. Unfortunately, maintaining the high reactivity of well-dispersed nZVI and avoiding their aggregation during application is challenging, which is not only ascribed to the aggregation caused by magnetic attraction forces and strong van der Waal among nZVI particles, but also owing to nZVI the oxidization of nZVI by dissolved oxygen and water even in an anoxic condition. Consequently, nZVI is always encapsulated or supported in/onto porous materials to surmount these shortages and to obtain better reactivity and reusability, which incidentally endows nZVI materials with the functionality of adsorption towards pollutants. Additionally, nZVI tends to be deactivated during reduction *via* the precipitation of Fe ions, which conversely promotes nZVI materials with a novel functionality in the removal or recovery of pollutants *via* co-precipitation. This mini-review surveys the recent advances of various environmental remediation approaches by nZVI based on its reductivity, such as direct reduction, adsorption–reduction, reduction–precipitation, adsorption–reduction–precipitation, electrolysis-assisted reduction, and bacterium-assisted reduction towards organic halides, nitrate, metal ions, radioactive heavy metals ions, *etc.*

However, there are still some challenges in the employing of nZVI in environmental remediation based on their reductivity:

(1) nZVI is susceptible to deactivation during reduction, which is mainly caused by the formation of Fe-containing deactivation layer *via* oxidation or/and the precipitation of Fe ions, leading to a low utilization efficiency of Fe^0^, poor performance in recycling, and an unsustainable reduction performance during application.

(2) The current reports on the application of nZVI based on their reductivity are usually in acidic conditions, thus lacking approaches that are specifically designed for the application in alkaline conditions. Meanwhile, there is also a lack of nZVI materials with higher reductivity for recalcitrant polyhalogenated aromatic hydrocarbons, which are widely distributed in waste circuit board dismantling materials and flame-retardant materials.

(3) It has been verified that the dehalogenation by various noble metal doped nZVI involves both a hydrogen atom transfer mechanism and an electron transfer mechanism. While, which of these two mechanisms has more advantage on dehalogenation efficiency and the maintenance of the reactivity of nZVI during application, which of these two mechanisms is more applicable for specific contaminants, and which of these two mechanisms can realize a higher utilization efficiency of nZVI, are all remained to be investigated.

In closing, to address these issues and pave the way for the practical application of nZVI, future research may focus on:

(1) Design and implement effective and environment-friendly protocols for the comprehensive utilization of nZVI. For instance, developing techniques for the reconfiguration and re-activation of deactivated nZVI in environmental remediation, and developing consecutive and synergistic remediation strategies for various contaminants.

(2) Exploring highly adaptable nZVI materials for the reduction applications under complex actual environmental conditions, especially applicable in alkaline conditions and multiple co-existing interferences.

(3) The ingredients in actual polluted bodies can be very complex and may possess various distinct hydrophilic and hydrophobic characteristics, which poses high requirements for nZVI materials on their functionalities and surface characteristics. Thus, regulating the surface chemical and electrochemical properties are crucial for designing nZVI materials applicating in remediation *via* adsorption–reduction and adsorption–reduction–precipitation.

(4) Integrating more functionality into nZVI materials and thus realize more novel applications, such as detoxification and enrichment of microplastics and various contaminants adsorbed on. Meanwhile, efficient remediation of polluted soils with metals/metalloids and organic halides is a very tricky issue and has attracted increasing attention, which calls for the development of a new remediation strategy that combines physical, chemical, and biological remediation techniques together in nZVI materials.

(5) In an effort to obtain a thorough remediation of organic contaminants, reduction alone may be not enough. As dissolving Fe(ii) generated during the reduction process can yet play a vital role in an extra Fenton oxidation process for the complete degradation of contaminant, future research may focus on the strategies of the combination of reduction and oxidation.

## Data availability

No primary research results, software or code have been included and no new data were generated or analysed as part of this review.

## Author contributions

Conceptualization, Mingyue Liu; writing – original draft preparation, Mingyue Liu; writing – review and editing, Gang Chen, Linli Xu, Yuyuan Ye and Zhicai He. All authors read and approved the final manuscript.

## Conflicts of interest

The authors declare no conflict of interest.
